# Effect of Royal Jelly on new bone formation in rapid maxillary expansion in rats

**DOI:** 10.4317/medoral.20581

**Published:** 2015-10-09

**Authors:** Fatih Özan, Bayram Çörekçi, Orçun Toptaş, Koray Halicioğlu, Celal Irgin, Fahri Yilmaz, Yasin Hezenci

**Affiliations:** 1 Abant Izzet Baysal University Faculty of Dentistry Department of Oral and Maxillofacial Surgery Bolu, Turkey; 2 Abant Izzet Baysal University Faculty of Dentistry Department of Orthodontics Bolu, Turkey; 3 Abant Izzet Baysal University Faculty of Medicine Department of Pathology Bolu, Turkey

## Abstract

**Background:**

The aim of this study was to evaluate the effects of long and short term systemic usage of royal jelly on bone formation in the expanded maxillary suture in a rat model.

**Material and Methods:**

Twenty eight Wistar albino rats were randomly divided into 4 equal groups: Control (C); Only Expansion (OE), Royal Jelly (RJ) group, Royal Jelly was given to rats by oral gavage only during the expansion and retention period; Royal Jelly plus Nursery (RJN) group, Royal Jelly was given to rats by oral gavage during their nursery phase of 40 days and during the retention period. After the 5 day expansion period was completed, the rats underwent 12 days of mechanical retention. All rats were sacrificed in same time. Histological examination was performed to determine the number of osteoclasts, number of osteoblasts, number of capillaries, inflammatory cell infiltration, and new bone formation.

**Results:**

New bone formation, number of osteoclasts, number of osteoblasts, and the number of capillaries in the expanded maxillary sutures were higher in the RJ and RJN groups than in the other groups. Statistical analysis also demonstrated that new bone formation and the number of osteoblasts was also highest in the RJN group.

**Conclusions:**

The systemic administration of Royal Jelly in conjunction with rapid maxillary expansion may increase the quality of regenerated bone.

**Key words:**Bone formation, rapid maxillary expansion, Royal jelly.

## Introduction

For the correction of malocclusions on the transverse dimension, mid palatal suture and maxillary expansion is a generally used procedure by orthodontists. It provides the increased transverse width at the apical base of the maxillary dental arch. The procedure has active and passive phases. In active phase, midpalatal suture widens by expansion appliances to disarticulate the two parts of the maxillary bone by rupture; and in passive phase, bone remodeling of the mid palatal suture occurs (1).

Some techniques that sustain exogenous forces transmitted as mechanical stresses to craniofacial sutures are a well-known controlling stimulus for modulating craniofacial growth in patients suffering from dentofacial deformities. Rapid maxillary expansion (RME) is one of these techniques. In the palate between the maxillary bones, an anatomical structure called the mid palatal suture contains secondary cartilage that is highly responsive to various mechanical forces (2,3). RME increases the posterior dentition width rapidly, which is followed by active bone formation in the mid palatal suture. Sutural mechanical strains, such as those caused during RME, trigger a biologic chain of events leading to new bone deposition in the mid palatal suture (4). Mesenchymal cells located on the inner side of the cartilaginous tissue proliferate and differentiate into osteoblasts when the suture is expanded (5). Many new materials especially antioxidants were used in studies and showed that antioxidants increases the osteoblastic activity and bone metabolism (6-8).

Royal jelly (RJ), a yellowish material excreted by the mandibular and hypopharyngeal glands of worker bees of the genus Apis mellifera, is a food essential for the longevity of the queen bee, and has been demonstrated to possess several pharmacological activities such as life-span-elongating (9), antifatigue (10), antiallergic (11), antitumor (12), antihypercholesterolemic (13), antihypertensive (14), and anti-inflammatory (15) effects. RJ has received particular attention because of studies reporting that it is a highly efficient antioxidant and has free-radical-scavenging capacity (16,17).

The aim of this experimental study was to evaluate the effects of long and short-term systemic usage of RJ on bone formation in the expanded maxillary suture in a rat model.

## Material and Methods

The current study was carried out in accordance with the guidelines for the use of laboratory animals and, all the experimental procedures were applied in the experimental animal facility of Abant İzzet Baysal University. The Institutional Animal Ethics Committee of Abant İzzet Baysal University approved the study.

Twenty-eight adult male Wistar albino rats, aged 12 weeks (average weight 200 ± 10 g), maintained under standard housing conditions (room temperature 25±3° C, humidity 60-65%, 12:12 h dark-light circle) and consuming a standard diet and water ad libitum during the procedure, were used for the study. The rats were housed separately in plastic cages.

The 28 rats were randomly divided into four groups of equal numbers (n= 7) as follows:

1. Control (C) group.

2. Only expansion (OE) group: a total of 17 days with retention period.

3. RJ given by oral gavage (100mg/kg) only during the expansion and retention period: a total of 17 days - (RJ).

4. RJ given by oral gavage (100mg/kg) during their nursery phase before expansion (a period of 40 days), and during the expansion and retention period: a total of 57 days - (RJN).

- Preparation of RJ

RJ was collected from Trabzon in Turkey was used throughout the experiments. It was suspended in sterile phosphate-buffered saline (PBS) at a concentration of 50 mg/ml. The supernatant of the RJ suspension was collected by centrifugation at 10 000 X G for 10 min.

- Expansion appliance and retention procedure

**Figure 1:**
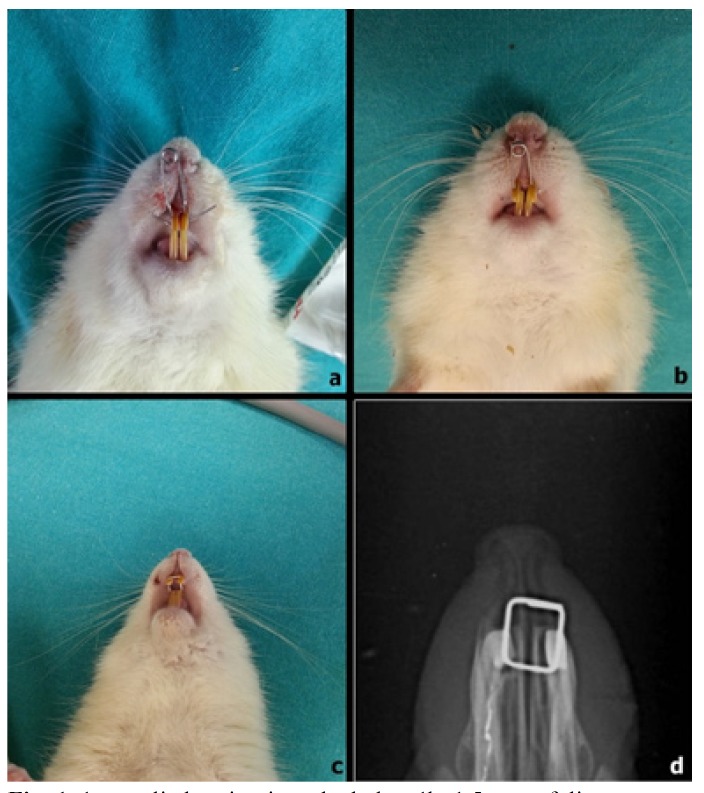
1a, applied spring into the holes; 1b, 1,5 mm of distance was provided after expansion period of 5 days; 1c, applied short lengths of rectangular wires; 1d, occlusal radiographs of the expanded premaxillary suture at the beginning of the retention.

The expansion appliance comprised helical springs fabricated from 0.012-inch, stainless steel wires. The helical springs were placed on a grid and activated on a single arm with pliers. The force was measured with a gauge (30 g), and the springs were not reactivated during the expansion period. The rats were anesthetized using an intraperitoneal injection of Brema®-Ketamin10%, 60-80 mg/kg (Bremer Pharma GMBH, Bremerhaven, Germany) and Alfazyne® 25, 8-10 mg/kg (Alfasan International B.V, Woerden, Holland). Expansion springs were attached to the maxillary incisor of all rats under anesthesia (Xylasine+ketamine combination, 0.5 ml/kg and 1ml/kg intramuscular, respectively). The prepared springs inserted into the holes that were drilled in both incisors at the same level, from the buccal side. The expansion period was 5 days in all the rats. In addition, occlusal radiographs were taken at the end of the expansion procedure in order to verify orthopedic expansion. It was observed that the maxillary suture was successfully expanded in all the expansion groups. In the OE, RJ, and RJN groups, the helical springs were removed after the expansion procedure, and a piece of rectangular retaining wire was inserted for retention (Fig. 1). These rats underwent 12 days of mechanical retention.

- Histological examination

As soon as the necessary procedure was completed for all groups, the animals were sacrificed in same time, and then their maxilla were dissected and placed in bottles containing 10% formalin. After fixation, the retaining wires were removed, and the maxilla was decalcified with 5% formic acid for 3 days. The decalcifying solutions were changed two or three times a day during decalcification.

The essential guide for section orientations were the maxillary incisors. The section was cut perpendicular to the sagittal plane and was determined by two points, one at the alveolar crest and the other 4 mm apically. This plane passed through the centre of the incisor crown at its gingival portion. The sections were rinsed, trimmed, and embedded in paraffin. The paraffin blocks were sliced into 5 µm thick sections. All subsequent analyses were performed by two experienced histopathologist blinded to the identity of the sections, and the average of the counts was obtained. The sample sections were routinely stained with hematoxylin-eosin and evaluated using a light microscope (Olympus CX41/DP25 Research System; Olympus Corporation, Tokyo, Japan).

Three histological sections from each animal were analyzed. The study and control groups were compared to establish the number of osteoclasts, osteoblasts, and capillaries, as well as the intensity of inflammatory cells, and new bone formation. The sections were rated as mild (+: 0-15 cells), moderate (++: 15-30 cells), or strong (+++ : >30 cells) for capillary intensity. However, new bone formation and inflammatory cell infiltration were qualitative features, and were evaluated in a subjective manner (mild: +; moderate: ++; or strong: +++), (+ = score1, ++ = score2, +++ = score3).

- Statistics

All statistical analyses were performed using the SPSS software package for Windows (version 15.0, SPSS, Chicago, Illinois). The difference was considered to be statistically significant at a *P* value of < .05.

Differences in the number of osteoclasts and capillaries among the four groups were evaluated with the use of the Kruskal-Wallis test, and the results were presented as the mean ± SD. Pair-wise comparisons were made with the use of the Mann-Whitney U test.

The parameters relating to the number of osteoblasts, inflammatory cell infiltration, and new bone formation were represented as the scores indicating intensities. The scores of the groups were compared using Fisher’s exact test.

## Results

- Histological findings

The following parameters were measured: number of osteoblasts, number of osteoclasts, number of capillaries, intensity of inflammatory cells, and new bone area (μm2).

- Number of osteoblasts.

**Figure 2:**
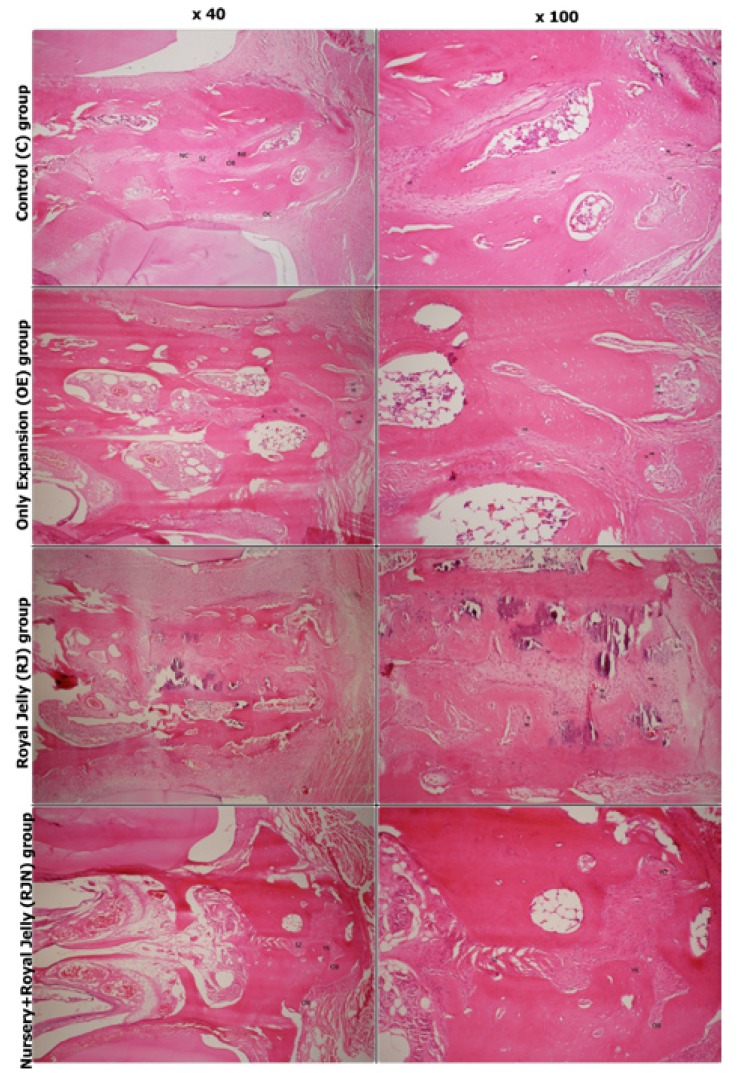
Hematoxylin and eosin staining photomicrographs from the study groups. OB indicates osteoblast; OC, osteoclast; NB, new bone; NC, new capillary; and SZ, premaxillary suture zone.

The difference between groups was statistically significant according to the number of osteoblasts. The histological findings revealed that the number of osteoblasts was significantly higher amongst the RJN group than the other groups (*P* < 0.001), whilst the lowest number of osteoblastic cells was found in control group. (Table  Table 1, Fig. 2).

**Table 1:**
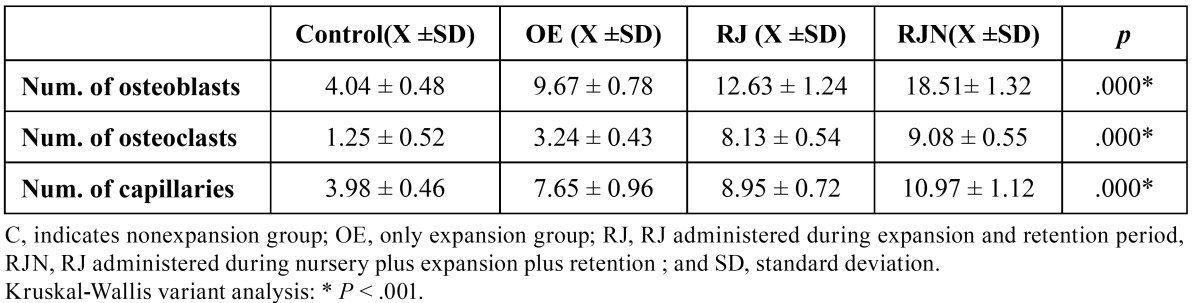
Effects of RJ on the number of osteoclasts, number of osteoblasts, and number of capillaries at the end of experimental period.

- Number of osteoclasts.

The lowest number of osteoclastic cells was found in the C (non expansion, control group) group (1.25 ± 0.52), which represented the physiological situation. The number of osteoclasts was significantly higher in the RJN and RJ groups than in the OE group (*P*<.001), indicating the increased activity. However, the Mann-Whitney U test showed that this parameter was not statistically significantly higher in the RJN group (8.13 ± 0.54) than in the RJ group (9.08 ± 0.55) (*P*˃ .001) ( Table 1 and  Table 2, Fig. 2).

**Table 2:**
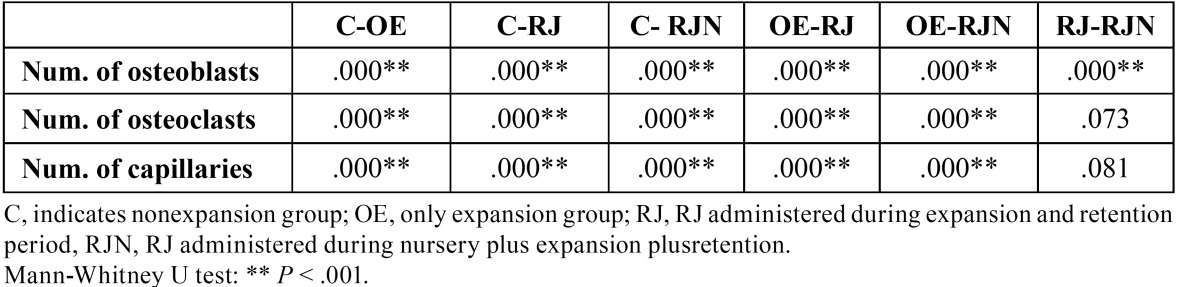
Pair-wise comparisons of the groups.

- New bone formation.

When the groups were compared for new bone formation, considerable differences were found amongst members of the RJN group compared to all of the other groups. The results showed that there was an increase in new bone formation in the RJN group that was significantly greater than in the other groups (*P*= .001), ( Table 3, Fig. 2). The RJ group also showed increased growth which exceeded that of the remaining groups.

**Table 3:**
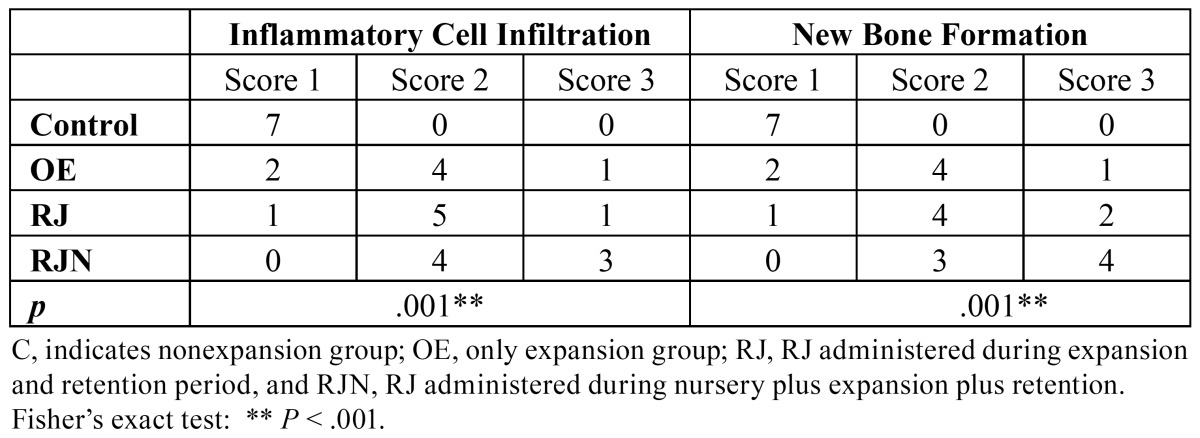
Effects of RJ on the inflammatory cell infiltration, and new bone formation scores indicate the number of subject animals representing that score.

- Number of capillaries.

The RJ and RJN groups showed a significant increase in the number of capillaries relative to the other groups (*P* < .001). However, the Mann-Whitney U test demonstrated that the number of capillaries was similar in the RJN and RJ groups ( Table 1 and  Table 2, Fig. 2).

- Intensity of inflammatory cells.

There was a significantly higher ratio of inflammatory cells in the RJN group than in the other groups.

## Discussion

Maxillary expansion occurs through a multi factorial adaptive response within the expanded maxillary suture. Mechanical expansion results in distortion at the sutural structure, inducing a biologic chain of events leading to osseous modeling which allows the suture to restore itself to its original architecture. At the end of this procedure, the clinical result is an increase in maxillary skeletal and dentoalveolar width.

Expansion of maxillary suture is associated with inflammation and ischemia, stimulating free radical oxidation like bone fracture (18). The effects of antioxidants on the early stage of bone healing have been reported (19,20). Free radicals generated in the osseous environment enhance osteoclast formation and bone resorption (21). Antioxidant therapies are especially promising since they inhibit osteoclastic activity and promote osteoblastic activity, and have been shown to be beneficial in suppressing the damaging effects of oxygen-free radicals on cells during bone healing (22,23).

Oxidative stress due to excessive production of reactive oxygen species (ROS) and/or impaired antioxidant defense mechanisms can result in adverse biologic effects on bone by inhibiting bone cell differentiation (24,25). Evidence indicates that ROS considerably affect the generation and survival of osteoblasts, osteoclasts and osteocytes, and play a role in bone resorption, with a direct contribution of osteoclast-generated super oxide to bone degradation, and also can directly promote osteoclast formation and activity (25,26). Thus, considering the detrimental effect of oxidants, various host-modulator agents such as antioxidants have been widely investigated for their ability to cope with the oxidant-related breakdown of hard tissues and for their possible role in promoting bone healing. New materials and methods were used and they were confirmed an effect on accelerating new bone formation at the expanded suture. Some of these studies showed that antioxidants affect bone metabolism, via an increase of osteoblastic activity (6,27).

RJ has been widely used by many of cultures for centuries since it has lots of benefits on human health. In the light of animal and clinical researches its widespread effects were shown. In the current study, systemic RJ intake and its effect on bone regeneration have been evaluated in expanded maxillary suture. Amount of RJ was determined by recent researches as 100mg/kg/day (28-30).

We have clearly demonstrated the stimulatory effects of RJ on bone regeneration in this area during maxillary expansion. Increased activity was determined by number of osteoblast, number of osteoclast and new bone formation. In RJ and RJN groups all of these parameters were shown significantly higher than control and only expansion groups. In conjunction with these results increased number of capillaries and intensity of inflammatory cells were also shown. This study is the first to present data indicating that royal jelly improves new bone formation in RME in rats. Effect of RJ may contribute to its antioxidant and bone stimulatory properties (28-30).

## Conclusions

These findings suggest that systemic administration of RJ can stimulate bone regeneration in an orthopedic ally expanded pre-maxillary suture, during expansion and retention periods. This stimulation increases with long-term use before expansion. RJ is used worldwide in many forms as nutritional supplement unlike the other antioxidants that used in studies to increase the bone metabolism during RME procedure. The RJ may become more appropriate than the other agents in clinical use due to this specialty.
